# Brown-Sequard syndrome caused by posterior full-endoscopic cervical discectomy: A case report

**DOI:** 10.1097/MD.0000000000042007

**Published:** 2025-04-11

**Authors:** Zhen-Yu Zhang, Tong Zhang, Longfei Gao, Xiao Liang, Xu Gao, Chun-Yang Meng

**Affiliations:** a Department of Clinical Medicine, Jining Medical University, Jining, Shandong Province, China; b Department of Spine Surgery, Affiliated Hospital of Jining Medical University, Jining, Shandong Province, China.

**Keywords:** Brown, endoscopic cervical discectomy, posterior full, Sequard syndrome, spinal cord injury

## Abstract

**Background:**

Posterior full-endoscopic cervical discectomy (PFECD) is an effective and safe technique for cervical radiculopathy. The primary complications of PFECD include temporary nerve root paralysis and dural rupture, while spinal cord damage is exceedingly rare. This study describes a rare case of Brown-Sequard syndrome (BSS) occurring following PFECD and investigates its potential etiologies and pathomechanisms associated with this procedure.

**Methods:**

Notes and images were reviewed and the relevant literature was analyzed.

**Results:**

A 50-year-old woman underwent PFECD for cervical radiculopathy. The patient reported substantial alleviation of radicular pain symptoms on the first postoperative day. On the third postoperative day, the patient exhibited acute-onset weakness in the left lower limb, along with diminished pinprick and temperature sensation in the right limb. Cervical spine magnetic resonance imaging demonstrated a newly developed T2 hyperintensity at the C5 spinal cord level. BSS was confirmed based on correlating imaging findings with clinical signs. Following the comprehensive treatment of rehabilitation and pharmacological therapy, the patient’s neurological deficits symptoms gradually improvement. At the 6-month follow-up, the patient’s symptoms resolved entirely, and the T2 hypersignal diminished markedly on repeat magnetic resonance imaging.

**Conclusion:**

This study represents the first case of BSS following PFECD. We emphasize that although the PFECD technique is safe and effective, meticulous surgical technique—particularly in foraminal decompression—is critical to avoid iatrogenic spinal cord injury.

## 1. Introduction

Recent advancements in spinal endoscopic procedures have led to the development of posterior full-endoscopic cervical discectomy (PFECD).^[1]^ PFECD employs a “keyhole” approach to achieve foraminal expansion through partial resection of the upper and lower facet joints, enabling effective nerve roots decompression. PFECD demonstrates equivalent clinical outcomes compared to anterior cervical discectomy and fusion in addressing cervical radiculopathy, with superior tissue preservation and accelerated postoperative recover.^[2]^ This operation has currently emerged as an effective method for treating cervical radiculopathy. Brown-Sequard syndrome (BSS) is a rare neurological disorder associated with hemisection spinal cord injury, classically presenting with ipsilateral motor paralysis, proprioceptive/vibratory deficits, and contralateral pain and temperature sensory loss.^[3]^ The primary complication of cervical percutaneous endoscopic surgery is temporary nerve root paralysis. The incidence of myelopathy resulting from percutaneous endoscopic surgery is exceedingly uncommon. We present a case of BSS following PFECD operation.

## 2. Case report

**Figure 1.**
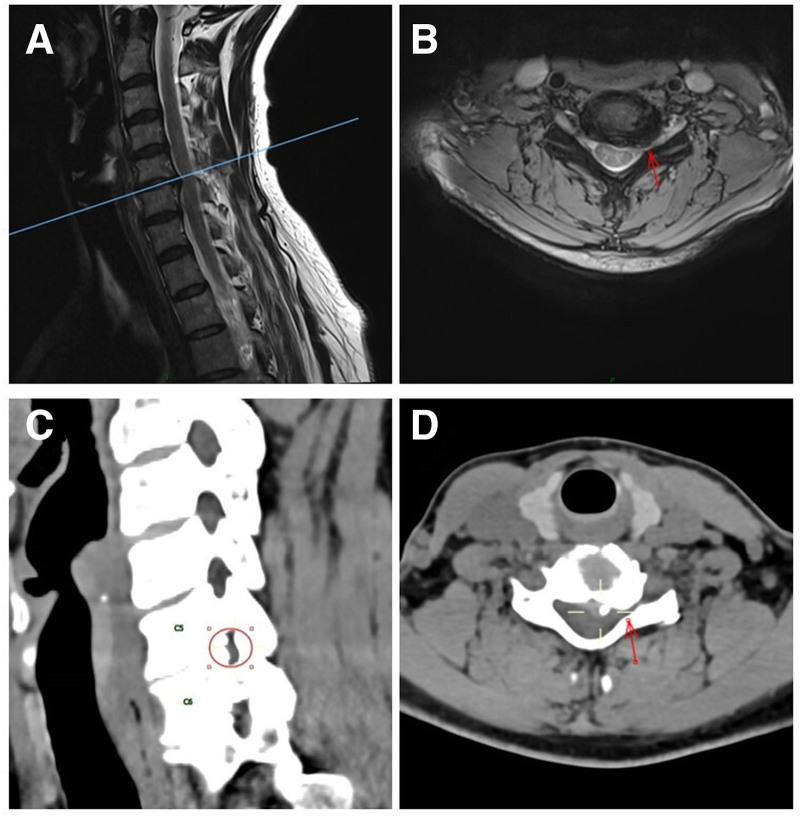
Preoperative T2W1 MRI imaging of the patient’s sagittal (A) and axial (B) reveals left-sided foraminal stenosis and the C5–C6 intervertebral disc herniation to the left rear. The preoperative CT examination (C, D) indicated stenosis of the 5 to 6 cervical foramen with uncovertebral joint hyperplasia. CT = computerized tomography, MRI = magnetic resonance imaging.

A 50-year-old woman with no significant medical history, including hypertension, diabetes mellitus, coronary artery disease, or cerebral infarction, presented with a 6-month history of persistent cervicobrachial pain radiating to the left upper arm and radial forearm, accompanied by left thumb paresthesia. Following 3 months of conservative treatment, the pain symptoms exacerbated, significantly impairing the patient’s daily life, with visual analog scale pain score of 7. Neurologic examination demonstrated: Left suprascapular tenderness; Grade 3/5 weakness in left biceps brachii and wrist extensors; Radial forearm and thumb allodynia; Positive left Eaton test, Spurling maneuver, and radial nerve stretch sign; and a negative bilateral Hoffmann sign. These findings correlated with C6 radiculopathy. Cervical magnetic resonance imaging (MRI) revealed left posterolateral disc herniation at C5–C6 with ipsilateral foraminal stenosis. Computerized tomography imaging further identified hypertrophic articular process and disc space narrowing at the same level. Dynamic cervical radiographs ruled out segmental instability or kyphotic deformity (Fig. 1).

**Figure 2.**
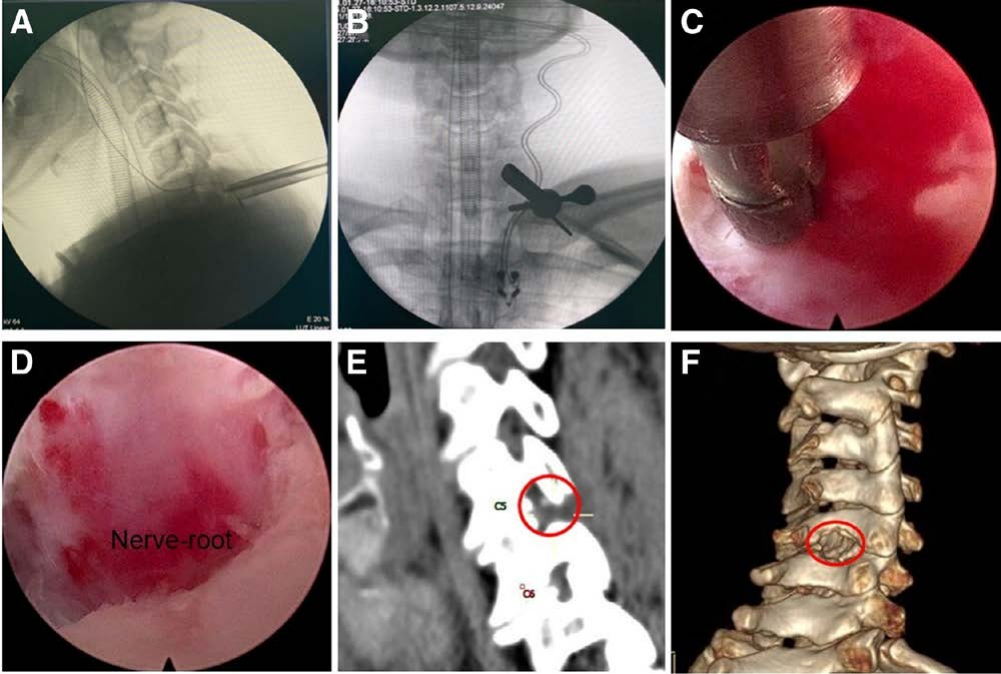
The K-wire was inserted through the safe operating area and anchored into the objective articular process, and serial dilators and endoscopic were established (A, B). Endoscopic radiofrequency electrocoagulation for hemostasis (C). The nerve root was completely decompressed (D). The sagittal view of CT (E) and three-dimensional reconstruction (F) demonstrate the enlargement of the intervertebral foramen. CT = computerized tomography.

The patient underwent PFECD surgery on the C5–C6 segments under general anesthesia. Intraoperative endoscopic visualization revealed left posterolateral disc protrusion at C5–C6 with C6 nerve root compression. Foraminal decompression was achieved via partial facetectomy and foraminal expansion, resulting in complete nerve root liberation. Intraoperative examination indicates that the left C6 nerve root had acquired adequate mobility, robust dural sac pulsation, and effective decompression. Intraoperative hemorrhage was 4 mL, and the procedure duration was 80 minutes. Continuous intraoperative neuromonitoring (somatosensory evoked potentials and motor evoked potentials) showed no signal abnormalities throughout the procedure (Fig. 2).

On the first postoperative day, the patient reported a substantial reduction in pain localized to the left side of the left neck and shoulder region, along with diminished numbness in the thumb, compared to the preoperative state, with a visual analog scale pain score of 2. The surgical incision showed no signs of redness, swelling, or exudation, and there were no apparent indications of infection.

On the third postoperative day, the patient exhibited BSS, manifesting as unilateral muscle weakness in the left lower limb, muscle strength at grade 2, and reduced pain and temperature sensation in the right limb, with pronounced involvement of the right lower limb. MRI demonstrated a new T2 hyperintense signal. Mannitol and dexamethasone were administered, leading to gradual symptom resolution. The patient was discharged on the fifth postoperative day and instructed to perform home-based functional rehabilitation exercises.

**Figure 3.**
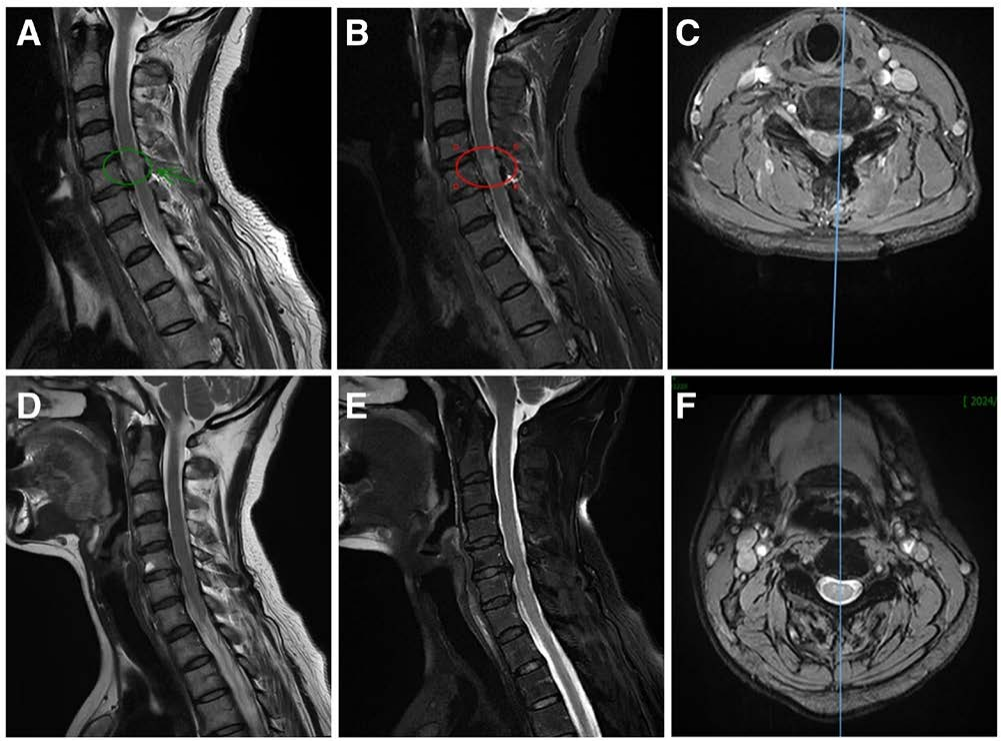
The patient underwent an MRI on the third postoperative day. Sagittal MRI images (A, T2W1; B, T2W1 STIR) revealed an abnormal T2 hyperintensity in the spinal cord at the cervical 5 vertebra level. The T2W1 STIR axial MRI (C) of the spinal cord revealed the aberrant signal bias to the left. MRI imaging at 6 months post-surgery. The T2 hyperintensity of the spinal cord at the level of the cervical 5 vertebrae was much lower than it had been before, as shown by sagittal MRI images (D, T2W1; E, T2W1 STIR) and axial MRI (F, T2W1 STIR). MRI = magnetic resonance imaging.

After a 6-month reevaluation, the neurologic examination showed no abnormalities. Sensory perception of pain and temperature in the right limb had fully recovered, and motor impairments in the left lower limb had completely resolved, with muscular strength rated at grade 5. The MRI demonstrated a significant reduction in the T2 hyperintense signal indicated a substantial reduction compared to the postoperative MRI scan (Fig. 3).

## 3. Discussion

Brown-Sequard first reported BSS syndrome in 1849.^[4]^ It is an uncommon neurological condition. This rare neurological disorder accounts for approximately 2% to 4% of traumatic spinal cord injuries.^[5]^ Moreover, blunt trauma can also cause BSS alongside direct penetrating injuries, such as aberrant disc herniation, spinal fractures, or gunshot wounds. BSS resulting from blunt trauma may be linked to spinal cord compression, vascular injury to the spinal cord, or stretching of the spinal cord.^[6]^ Besides acute BSS, BSS has also been documented in patients with other nontraumatic spinal diseases, such as arachnoid cysts, spinal cord cavities, tumors, epidural hematomas, and meningococcal myelitis.^[7,8]^

Postoperative BSS of the cervical spine is recorded in a restricted number of case reports, primarily subsequent to posterior cervical laminectomy.^[9]^ The occurrence of PFECD resulting in postoperative BSS is not presently reported. The occurrence of BSS correlates with the severity of preoperative spinal cord compression; greater spinal cord compression increases the likelihood of BSS during spinal cord decompression. The predominant complication following percutaneous endoscopic discectomy is temporary nerve palsy, and spinal cord damage subsequent to posterior cervical fusion is quite uncommon.^[10]^ Yang et al^[11]^ reported a case of worsening neurologic function in the contralateral lower extremity after PFECD, with gradual recovery of spinal cord injury symptoms after 3 months of conservative treatment, which may have been due to intraoperative spinal cord traction. Korinth et al^[12]^ reported a case of mild paralysis of the lower extremities after posterior foraminotomy with complete recovery within 3 weeks. Although most partial spinal cord injuries are transient and reversible, spinal cord injuries have a potentially high risk of poor prognosis and deserve constant vigilance by clinicians.

We further reviewed and analyzed the risk factors causing BSS. We concluded that the occurrence of BSS after PFECD may be related to intraoperative radiofrequency electrocoagulation stimulation for hemostasis, spinal cord ischemia-reperfusion injury, and spinal cord nerve traction injury. Radiofrequency (RF) electrodes serve as standard hemostatic tools in spinal endoscopic surgery. The mechanism involves ionic dissociation in saline to generate a plasma layer, enabling tissue ablation and coagulation through controlled thermal energy.^[13]^ The use of radiofrequency may lead to 2 types of thermal injuries: contact thermal damage to neural structures from activated RF probes; indirect injury: epidural temperature elevation during prolonged RF activation.^[14]^ RF-associated complications—including chondrolysis, osteonecrosis, and peripheral neuropathy—are well-documented in arthroscopic procedures.^[15]^ In general, the small-diameter controllable bipolar radiofrequency tip has low output power and weak thermal penetration, which, when used with reasonable operation, does not cause direct thermal damage to the nerves. However, when it is used repeatedly for long periods of time, it may still cause the accumulation of heat, which increases the risk of thermal injury to the nerves.^[16]^ Therefore, we suggest that when performing hemostasis on the dural surface, it is necessary to cooperate with the saline perfusion cycle, pre-tune the radiofrequency power down, and avoid a single coagulation time of more than 2 seconds. These measures can, to some extent, help prevent neurothermal injury.

White cord syndrome (WCS), or spinal cord ischemia–reperfusion injury, occurs when sudden reperfusion post-decompression triggers oxygen-free radical overproduction and inflammatory cytokine surge (TNF-α, IL-1β), disrupting the blood–spinal cord barrier.^[17]^ The incidence and severity of WCS are closely related to the duration of ischemia, the extent of ischemic tissue, and the oxygen demand of the affected tissue. WCS typically manifests within 3 hours post-decompression; however, delayed presentations (24–72 hours) may occur in patients with chronic vascular comorbidities. Yen et al^[18]^ reported a case of delayed WCS, which may be related to endothelial damage and atherosclerosis caused by chronic hypertension and lower-than-normal spinal cord blood perfusion. Intraoperatively, somatosensory evoked potentials and motor evoked potentials are essential for the early detection of spinal cord injury, compression, or ischemia. Postoperatively, prompt recognition of spinal cord injury and initiation of high-dose methylprednisolone within 8 hours of injury are critical. Steroids exert neuroprotective effects by upregulating anti-inflammatory mediators and reducing oxidative stress, thereby enhancing endogenous cell survival.

Due to the relatively small operating space in cervical endoscopy, surgical maneuvers are often directed at overstretching the nerve roots in order to accommodate nucleus pulposus removal. We suggest that the main purpose of PEDCF should be to decompress the intervertebral foramina without blindly pursuing nucleus pulposus removal; at the same time, the precise design of the access direction should avoid difficulties in nucleus pulposus removal caused by the obstruction of the nerve root and minimize the pulling of the nerve root.

## 4. Conclusion

This study represents the first case of BSS following PFECD. We emphasize that although the PFECD technique is safe and effective, meticulous surgical technique—particularly in foraminal decompression—is critical to avoid iatrogenic spinal cord injury.

## Acknowledgments

The authors would like to thank our department colleagues and these patients for their dedication, and those patients or their next of kin had signed the informed consent forms.

## Author contributions

**Conceptualization:** Zhen-Yu Zhang, Chun-Yang Meng.

**Project administration:** Zhen-Yu Zhang.

**Writing – original draft:** Zhen-Yu Zhang, Tong Zhang.

**Writing – review & editing:** Longfei Gao, Xiao Liang, Xu Gao, Chun-Yang Meng.
